# Induction of Ectopic Retina-Like Tissue by Transgenic Expression of *neurogenin*

**DOI:** 10.1371/journal.pone.0116171

**Published:** 2015-01-30

**Authors:** Run-Tao Yan, Li He, Wenjie Zhan, Shu-Zhen Wang

**Affiliations:** Department of Ophthalmology, University of Alabama at Birmingham School of Medicine, Birmingham, Alabama, United States of America; Universidade Federal do Rio de Janeiro, BRAZIL

## Abstract

Degeneration of retinal neurons is an underlying cause of several major types of blinding diseases, and effective therapies remain to be developed. The suppositive strategy of repopulating a degenerative retina with new cells generated onsite faces serious challenges, because the mammalian retina seems to lack the ability to regenerate itself or replace its lost neurons. We investigated the possibility of using a transcriptional factor with proneural activities to reprogram ocular tissue with regenerative capability to give rise to retinal cells. Transgenic mice were generated with DNA constructs that targeted the expression in the retinal pigment epithelium of proneural gene *neurogenin1* from the promoter of *Bestrophin1*, or *neurogenin3* from *RPE65* promoter. Here we report the presence of ectopic retina-like tissue in some of the transgenic mice, young and aged. The ectopic retina-like tissue contained cells positive for photoreceptor proteins Crx, recoverin, red opsin, and rhodopsin, and cells positive for proteins that label other types of retinal neurons, including AP2α and Pax6 for amacrine cells, Otx2 for bipolar cells, and Brn3A for ganglion cells. The retina-like tissue often co-existed with darkly pigmented tissue positive for RPE proteins: cytokeratin 18, Otx2, and RPE65. The ectopic retina-like tissue was detected in the subretinal space, including two retinae co-existing in the same eye, and/or in the optic nerve or in the vicinity of the optic nerve head. On rare occasions, it was detected in the choroid and in the vicinity of the ciliary body. The presence of ectopic retina-like tissue in the transgenic mouse supports the possibility of inducing retinal regeneration in the mammalian eyes through gene-directed reprograming.

## Introduction

The neural retina is a thin tissue in the back of the eye and is responsible to initiate vision when the light enters the eye. In the retina, photoreceptor cells capture photons and generate electrophysiological signals. The signals are modulated by interneurons comprised of horizontal, bipolar, and amacrine cells and relayed by retinal ganglion cells to the brain for visual perception. Immediately outside the neural retina lies a single-layered, darkly pigmented, non-neural tissue—the retinal pigment epithelium (RPE), which plays important roles in retinal physiology. Retinal degeneration and RPE atrophy are the main causes of many common forms of visual loss, including retinitis pigmentosa, age-related macular degeneration, diabetic retinopathy, and glaucoma. The low quality of life of people with visual loss has spurred research interests in a wide range of potential therapeutic approaches, including retinal regeneration.

Naturally occurring regeneration from retinal stem cells would be ideal for producing new cells to repopulate a retina inflicted with degeneration. However, the presence of retinal stem cells in adult mammals remains elusive [1,2]. The reported retinal stem cells in the ciliary margin of the mouse eye [3,4] have been contested [5,6]. This has stimulated expanding the search for stem-like cells for retinal regeneration into other ocular tissues/cells.

One of the ocular tissues being investigated is the RPE. Anatomically, the RPE would be a convenient source of new cells to repopulate a degenerative retina. Developmentally, the RPE and the retina share a common origin, the optic vesicle. Biologically, mammalian RPE, including that of human, is capable of two cellular events: proliferation and plasticity. It has been shown that in a mature eye a small population in the periphery RPE maintain mitotic activities [7]. The majority of RPE cells, while normally quiescent, can reenter the cell cycle to proliferate under certain physical and pathological conditions, such as retinal detachment [8–10], physical stimulation [11], and retinal injury or degeneration [12–14]. The proliferative response may result in RPE regeneration [15–19] and may lead to proliferative retinopathy when progeny cells transdifferentiate into cells with tractional force causing retinal detachment [20]. Experimentally, RPE-to-retina transdifferentiation can occur in RPE culture derived from very young mouse embryos [21,22] and in vivo by gain- or loss-of function of genes involved in the fate choice of RPE vs. retina [23–29]. Nonetheless, this RPE-to-retina transdifferentiation capability seems lost in differentiated RPE, and no RPE remains after the transdifferentiation in vivo, thus diminishing its prospect as a potential approach to eliciting retinal regeneration to replace lost cells.

A gene-directed reprograming of differentiated RPE to serve as a potential source of new retinal neurons has been tested with chick and mammalian cells. *Neurogenin1* (*ngn1*) and *ngn3*, members of the *neurogenin* subfamily of the basic Helix-Loop-Helix family of transcription factors and homologous to the *Drosophila* proneural gene *atonal*, can efficiently reprogram differentiated RPE cells of embryonic chick to develop into cells displaying molecular, morphological, and physiological properties of young photoreceptor cells, with small fractions of the product cells expressing markers of other types of retinal neurons [30]. Similar reprogramming also occurs in primary RPE cell cultures of juvenile pig and postnatal mouse and in human RPE cell line cultures [31]. In transgenic mice generated with DNA expressing *ngn1* from the promoter of *Bestrophin1* (P_VMD2_-ngn1) or *ngn3* from the promoter of *RPE65* (P_RPE65_-ngn3), photoreceptor-like cells were detected in the subretinal space in both young and aged animals [32].

An even more audacious and elusive aim is to regenerate a retina in the mammalian eye. In Drosophila, targeted misexpression of *eyeless* (*Pax6*) produces ectopic eyes on the wings, the legs, and the antennae [33]. This has fueled attempts to elicit ectopic eye development in vertebrates using genes with key roles in inducing eye formation during development. Studies have shown that *Pax6* [34], *Six3* [35], and *Six6* [36] each can promote the generation of ectopic optic vesicles or relatively normal looking eyes in restricted regions of the embryonic head in Xenopus (*Pax6* and *Six6*) and medaka fish (*Six3*). Ectopic expression in the hindbrain-midbrain area of *Six3* from *Pax2* promoter leads to the formation of ectopic optic vesicle-like structures in about ½ of the transgenic mice [37]. Whether the optic vesicle-like structures remained or existed in adult mice is unknown.

To determine whether the P_VMD2_-ngn1 and P_RPE65_-ngn3 designs in generating transgenic mice [32] could promote the generation of retina-like tissue, we examined the eyes of the transgenic mice with histology and immunohistology. Here we describe the presence of ectopic retina-like tissue in the choroid, near the ciliary body, in the optic nerve, as well as in the native locale of the retina—the subretinal space and/or expanded subretinal space. The retina-like tissue was present in young and adult mice. Our data points to a possible approach to retinal regeneration in the mammalian eye.

## Materials and Methods

### Generation of transgenic mice

All experimental procedures involving the use of animals adhered to the recommendations in the Guide for the Care and Use of Laboratory Animals of the National Institutes of Health and were approved by the Institutional Animal Care and Use Committee of the University of Alabama at Birmingham (Permit Number: 140909197). All efforts were made to minimize suffering. Euthanization was carried out with CO_2_ inhalation and cervical dislocation (young animals).

Transgenic mice were generated as previously described [32]. Briefly, the sequence (−585 to +38) of mouse *Bestrophin1* promoter (P_VMD2_) and the sequence (−737 to 0) of mouse *RPE65* promoter (P_RPE65_) were PCR amplified, cloned into pGEM-T, and sequence verified. P_RPE65_ (or P_VMD2_) was inserted into vector pCI replacing P_CMV_ to drive transgene expression in the RPE. The coding sequences of human *ngn1* and *ngn3* were RT-PCR amplified and cloned into pGEM-T. After sequence verification, the coding regions were linked with P_VMD2_ and P_RPE65_ to generate pCI-P_VDM2_-ngn1 and pCI-P_RPE65_-ngn3, respectively, which were used to create potential founders at the University of Alabama at Birmingham Transgenic Facility. Transgenic animals were identified by PCR genotyping with DNA from tail snips.

### Immunohistochemistry

Tissue preparation, cryosectioning, and immunohistochemistry were performed as previously described [32]. Briefly, eyes were enucleated after euthanization, fixed, and processed for cryosectioning. Cryosections of 10–12 μm were collected onto glass slides. Standard procedure of immunohistochemistry was followed. Primary, monoclonal antibodies included those against Brn3A (1:200 dilution; Chemicon), cytokeratin 18 (1:200 dilution; Sigma), endothelial cells (1:200 dilution; MAB A10-33/1, ABCam), glutamine synthetase (1:200 dilution; MAB302, Millipore), Pax6 (1:100 dilution; supernatant, Developmental Hybridoma Bank), rhodopsin (1:200 dilution; Chemicon), and RPE65 (1:200 dilution; Novus). Primary, polyclonal antibodies included those against AP2α (1:200 dilution; Bioworld Technologies), cellular retinaldehyde binding protein (1:200 dilution; Proteintech Group), Crx (1:300 dilution; Santa Cruz), glial fibrillary acidic protein (1:100 dilution; Sigma), Otx2 (1:200 dilution; AB9566, Millipore), recoverin (1:700 dilution; Chemicon), and red opsin (1:200 dilution; Chemicon). Secondary antibodies used included Alex 594-conjugated goat-anti-rabbit, Alex 594-conjugated goat-anti-mouse, Alex 488-conjugated goat-anti-rabbit, and Alex 488-conjugated goat-anti-mouse (1:200 dilutions; Invitrogen). Specificity of the immunostaining was confirmed by the anatomical locations of positive cells in cross-sections of the mouse eye.

## Results

The underlying theme of the creation of P_VMD2_-ngn1 and P_RPE65_-ngn3 transgenic mice [32] was to use the proneural activities of *ngn1* and *ngn3* to channel RPE’s proliferation and plasticity towards de novo generation of retinal neurons, particularly photoreceptor cells, as observed with cultured RPE cells derived from chick embryos [38]. The promoters of *Bestrophin1* and *RPE65* were used because they are primarily, although not exclusively, expressed in differentiated RPE cells [39,40]. The design also considered temporal down-regulation of the transgene. Activating the transgene expression in RPE cells would result in the expression of genes that initiate a cascade of events leading to photoreceptor differentiation. As the cell switching its identity, the expression of RPE genes, including the transgene itself from the RPE promoter, would be suppressed. This would emulate the transient *ngn1* and *ngn3* expression during neurogenesis observed in the chick retina. In approximately 60% of offspring from ~50% of founders, there are photoreceptor-like cells in the subretinal space [32]. The reason for the incomplete penetrance remains unclear. Attempts to correlate phenotypic manifestation with transgene expression proved to be in vain, with the design of targeting for a transient transgene expression as one of the potentially multiple causes for the unfruitful effort.

### Retina-like tissue in the subretinal space

**Figure 1:**
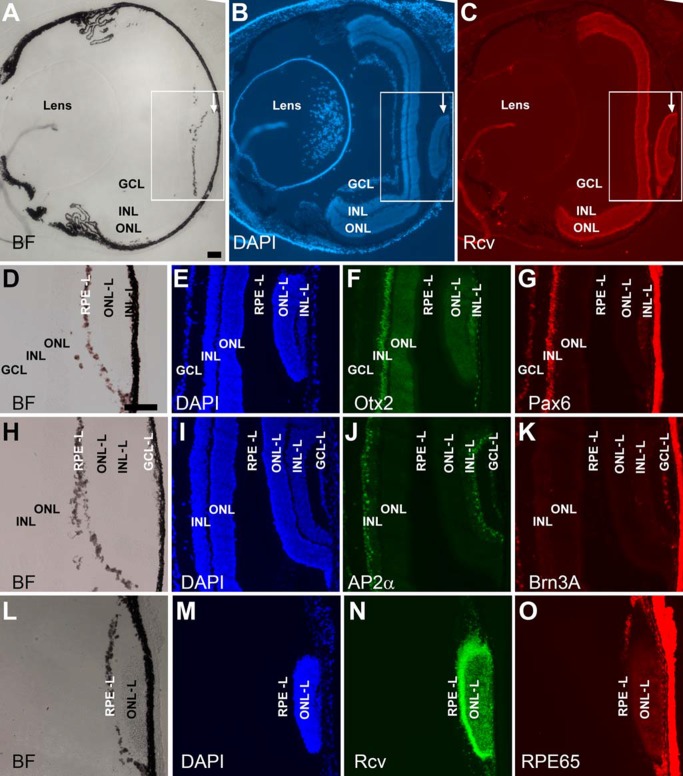
Retina-like tissue in the subretinal space of a 2-week-old P_RPE65_-ngn3 mouse. A–C: Low magnification images to show the relative position of retina-like tissue (arrow) in a cross-section of the eye, with the anterior to the left and the posterior to the right. A: Bright-field (BF) view. B: Epi-fluorescence of DAPI staining of the nuclei. C: Epi-fluorescence of anti-recoverin (Rcv) immunostaining to mark photoreceptor cells. Boxes outline the corresponding regions in A–C, or similar locations on serial sections (D–O). D–G: A serial section under bright-field view (D), after DAPI nuclear staining (E), and after double immunostaining for Otx2 (in green, F) to identify bipolar cells and for Pax6 (in red, G) to label amacrine cells in the INL and in the GCL (misplaced amacrine). H–K: A serial section under bright-field view (H), with DAPI nuclear staining (I), and with double immunostaining for AP2α (in green, J) to label horizontal (albeit weakly) and amacrine cells in the INL and in the GCL (misplaced amacrine), and for Brn3A (in red, K) to identify a subset of ganglion cells. L–O: A serial section under bright-field view (L), with DAPI nuclear staining (M), and with double immunostaining for photoreceptor protein recoverin (in green, N) and for RPE protein RPE65 (in red, O). RPE-L, RPE-like; ONL-L, outer nuclear layer-like; INL-L inner nuclear layer-like; GCL-L, ganglion cell layer-like. Scale bar (100 μm) in A also applies to B, C. Scale bar (100 μm) in D also applies to E–O.

DAPI staining of retinal cross-sections showed the presence in some of the P_VMD2_-ngn1 and P_RPE65_-ngn3 mice of extra piece of tissue with histological nuclear demarcation typical of the retina. In the eye of a 2-week-old P_RPE65_-ngn3 mouse, a small piece of such tissue was detected at the posterior region (Fig. 1B, E, I, M). To examine whether the tissue was indeed retina-like, we carried out immunostaining of serial sections with antibodies that recognize distinctive proteins of cells in retina’s three nuclear layers: the outer nuclear layer (ONL), the inner nuclear layer (INL), and the ganglion cell layer (GCL). Like the retina, the thickest nuclear layer of the extra tissue was labeled by antibody against recoverin, a protein involved in phototransduction in photoreceptors of the ONL (Fig. 1C, N). The next layer contained cells positive for proteins typically expressed in INL neurons, including Otx2 in bipolar cells (Fig. 1F), Pax6 in amacrine cells (Fig. 1G), and AP2α in horizontal cells weakly and in amacrine cells in the INL and misplaced ones in the GCL (Fig. 1J). The thinnest layer of the retina-like tissue contained cells labeled by antibody against ganglion cell protein Brn3A (Fig. 1K). The three layers of the retina-like tissue were subsequently referred to as ONL-like, INL-like, and GCL-like, respectively.

The retina-like tissue was often associated with melanized or pigmented tissue (Fig. 1A, D, H, L). To examine whether the tissue was RPE-like, immunostaining with specific antibody against RPE65, a protein primarily expressed in the RPE [40], was carried out. RPE65^+^ cells were identified in the ectopic, melanized tissue (Fig. 1O).

Notably, unlike the retina whose ONL resides proximal to the RPE, the GCL-like layer of the retina-like tissue localized proximal to the RPE. This reversed tissue polarity has been observed with the retina generated from the RPE through transdifferentiation [38, 41, 42].

**Figure 2:**
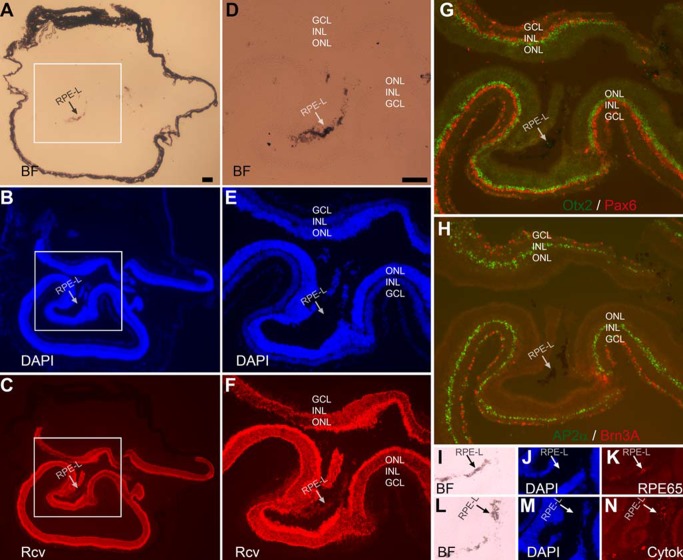
Extra retina in a 2-month-old P_RPE65_-ngn3 mouse. A–C: Low magnification images to show the relative positions of the retinae in a cross-section of the eye, with the anterior facing up. A: Bright-field (BF) view. B: DAPI staining of the nuclei. C: Anti-recoverin (Rcv) immunostaining to mark photoreceptor cells. D–F: High (2.5X) magnification of the boxed region in A–C to show the well-defined 3 nuclear layers of two retinae coexisting at the region. G: Double immunostaining of a serial section for Otx2 (in green) to mark RPE cells and bipolar cells, and for Pax6 (in red) to mark amacrine cells in the INL and in the GCL (misplaced amacrine). H: Double immunostaining of a serial section for AP2α (in green), a marker for horizontal cells and amacrine cells in the INL and misplaced ones in the GCL, and for Brn3A (in red), a marker for a subset of ganglion cells. I–N: Immunostaining of serial sections for RPE65 (K) and for cytokeratin 18 (N), markers of RPE cells. Bright-field (I, L) and DAPI staining (J, M) were provided to show the histology. RPE-L: RPE-like; GCL: ganglion cell layer. Scale bar (100 μm) in A also applies to B, C. Scale bar (100 μm) in D also applies to E–N.

Eyes of adult mice were then analyzed to examine whether they might contain retina-like tissue, regardless of the tissue either having survived into adulthood or being generated in adulthood. In a 2-month-old P_RPE65_-ngn3 mouse, two retinae of similar sizes coexisted in the same eye (Fig. 2). Each of the retinae showed the 3 distinctive nuclear layers (Fig. 2B, E). The ONLs in both retinae were positive for recoverin (Fig. 2C, F), and the INLs positive for AP2α (Fig. 2H), Otx2 (Fig. 2G), and Pax6 (Fig. 2G). The GCLs were positive for ganglion protein Brn3A (Fig. 2H), as well as for AP2α (Fig. 2H) and Pax6 (Fig. 2G) of misplaced amacrine cells. Like in the case of small piece of retina-like tissue (Fig. 1), there was melanized tissue (Fig. 2A, D, I, L) between the two retinae. Immunostaining for RPE proteins showed positive signals with antibodies against RPE65 (Fig. 2K), cytokeratin 18 (Fig. 2N), and Otx2 (Fig. 2G; arrow). Otx2, while being expressed in bipolar cells in a mature retina, is also expressed in the RPE, where it plays important roles in RPE differentiation and in maintaining RPE properties. Retina-like tissue in the subretinal space was less frequently observed in mice older than 5 months of age, even though we did detect its presence in a 13-month-old P_VMD2_-ngn1 mouse that also contained substantial amount of darkly pigmented tissue in the optic nerve (see next subsection). We estimated that ~10% of the transgenic mice displaying photoreceptor-like cells in the subretinal space [32] contained retina-like tissue, however small or large in sizes, co-existing with the retina in the same eye.

**Figure 3:**
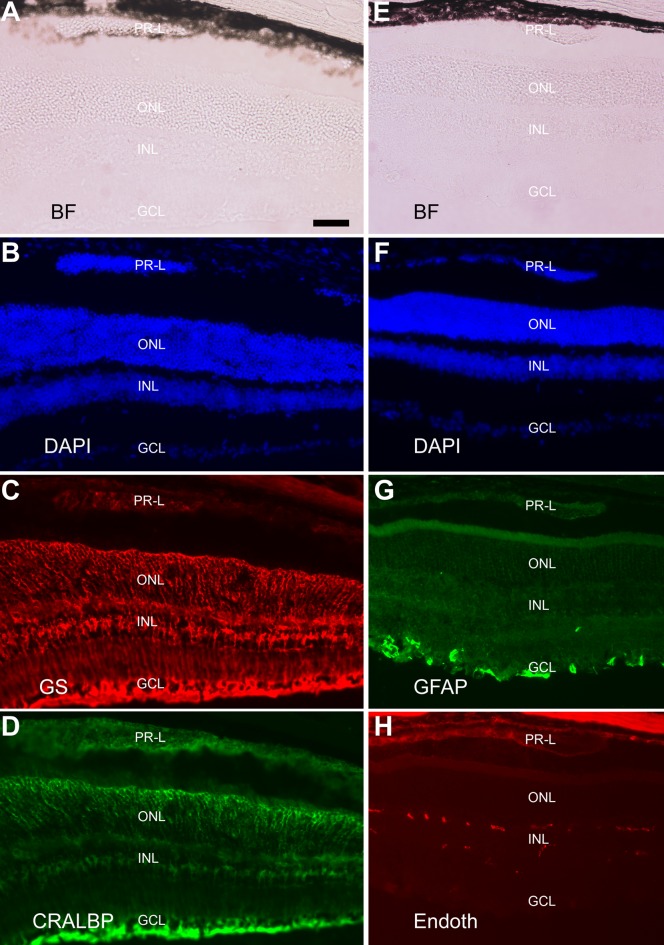
The presence of Müller glia, astrocytes, and endothelial cells of blood vessels in the retina containing photoreceptor-like (PR-L) cells in the subretinal space. A–D: Bright-field view (A), DAPI staining of the nuclei (B), and double-immunostaining for Müller proteins GS (C) and CRALBP (D) of a cross-section of the eye from a 2.5-month-old P_RPE65_-ngn3 mouse. E–H: Bright-field view (E), DAPI staining of the nuclei (F), and double-immunostaining for astrocyte protein GFAP (G) and endothelia cells (Endoth) with A10-33/1 (H) of a cross-section of the eye from a 5-month-old P_RPE65_-ngn3 mouse. Scale bar, 50 μm, applies to all panels.

**Figure 4:**
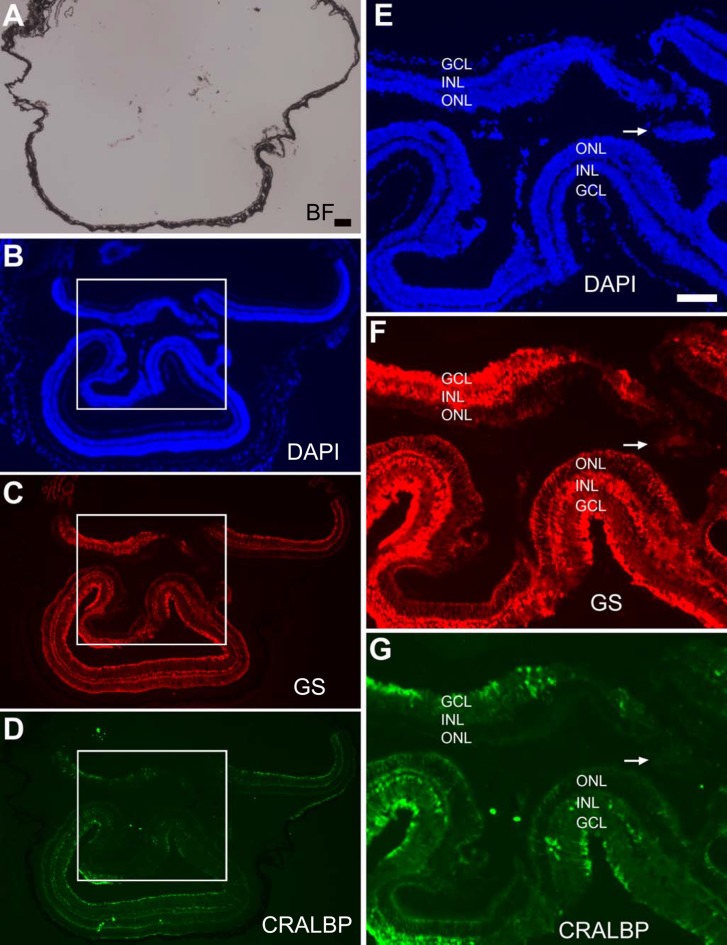
Distribution of Müller glia in an eye with two major retinae. A–D: Bright-field view (A), DAPI staining of the nuclei (B), and double-immunostaining for Müller proteins GS (C) and CRALBP (D) of a serial section of the eye shown in Fig. 2 (2-month-old P_RPE65_-ngn3 mouse). CRALBP^+^ cells were more or less evenly distributed in regions proximal to the RPE, whereas the distribution became patchy in the centrally located region (boxed). E–G: High (2.5X) magnification of the boxed region in B–D, respectively. Arrow points to a piece of retinal tissue with no detectable GS and CRALBP protein. Scale bars (100 μm) in A also applies to B–D and that in E also applies to F, G.

**Figure 5:**
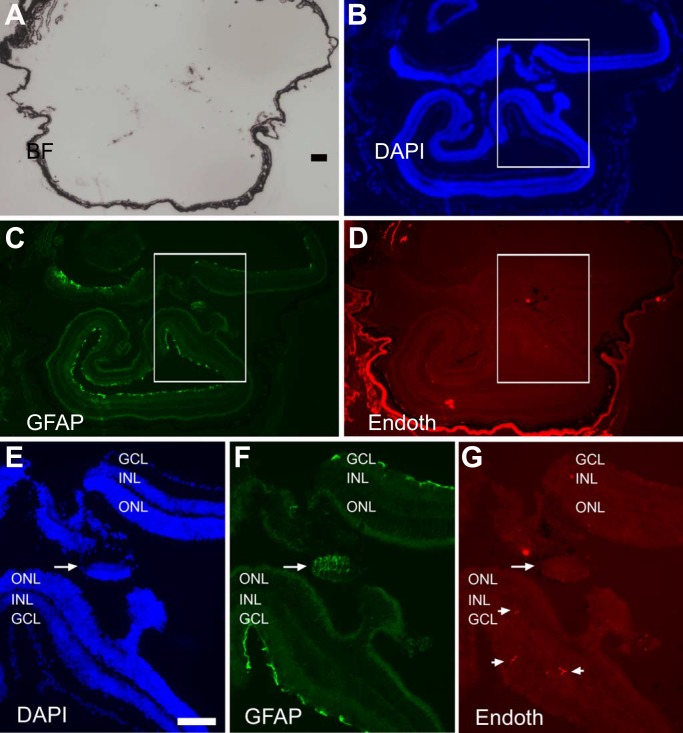
Presence of astrocytes and diminished endothelial cells of blood vessels in an eye with two major retinae. A–D: Bright-field view (A), DAPI staining of the nuclei (B), and double-immunostaining for GFAP (C) and endothelia cells (Endoth) with A10-33/1 (D) of a serial section of the eye shown in Fig. 2 (2-month-old P_RPE65_-ngn3 mouse). E–G: High (2.5X) magnification of the boxed region in B–D, respectively. Arrow points to a piece of retinal tissue with increased GFAP expression. Short arrows point to the scantily present endothelial cells. Scale bars (100 μm) in A also applies to B–D and that in E also applies to F, G.

The presence of retinal-like tissue in the subretinal space promoted the question whether there were Müller glia, astrocytes, and blood vessels in the retina-like tissue and in the retina. Immunohistological staining was performed with specific antibodies against glutamine synthetase (GS) expressed in Müller glia, cellular retinaldehyde binding protein (CRALBP) expressed in Müller glia (as well as in the RPE), and glial fibrillary acidic protein (GFAP) expressed in astrocytes and activated Müller glia, and antibody A10-33/1 against endothelial cells of blood vessels. In eyes with a relatively small amount of photoreceptor-like cells present in the subretinal space, CRALBP^+^ and GS^+^ Müller glia appeared normally present and distributed (Fig. 3 A–D), and so were GFAP^+^ astrocytes and A10-33/1^+^ endothelial cells (Fig. 3 E–H). Abnormalities were detected in the eye with two major retinae (shown in Fig. 2). In this eye, CRALBP^+^ and GS^+^ Müller glia appeared normally present at the regions proximal to the RPE, but became patchy, with areas lacking positive cells, in the centrally located region (Fig. 4). In this region, a small piece of retinal tissue (arrow, Fig. 4 E–G) conspicuously lacked CRALBP and GS immunoreactivity. Notably, this region on a serial section showed strong anti-GFAP immunoreactivity (arrow, Fig. 5E, F), an indication of Müller glia activation. In both of the two retinae, GFAP^+^ astrocytes remained present, whereas A10-33/1^+^ endothelial cells’ presence became scant (Fig. 5).

### Retina-like tissue in the optic nerve and the vicinity of the optic nerve head

**Figure 6:**
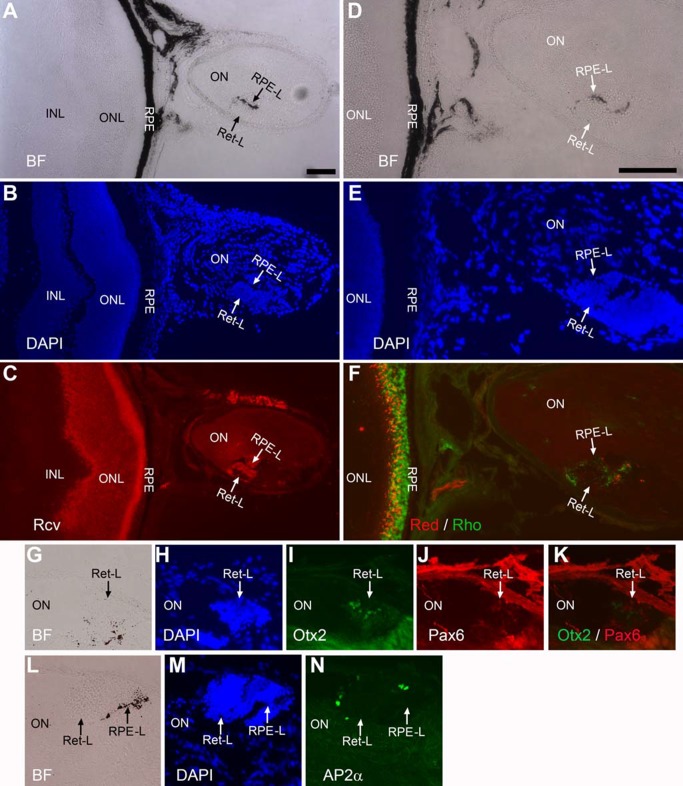
Retina-like tissue in the optic nerve (ON) in a P19 P_RPE65_-ngn3 mouse. A–C: Bright-field (A), DAPI staining (B), and immunohistochemistry with anti-recoverin (Rcv) immunostaining to mark photoreceptor cells of a posterior region of the eye. D–F: Bright-field (D), DAPI staining (E), and double immunostaining for rhodopsin (Rho) and for red opsin (Red) of a serial section of the region. G–K: Bright-field (G), DAPI staining (H), and double immunostaining for a bipolar cell marker Otx2 (I) and for an amacrine cell marker Pax6 (J) of a serial section of the region. K: A merge of I and J to show the non-overlapping localization of Otx2^+^ (in green) and Pax6^+^ (in red) cells in the retinal-like tissue in the optic nerve. L–O: Bright-field (L), DAPI staining (M), and immunostaining for AP2α (N), a marker for horizontal cells and amacrine cells of a serial section of the region. Scale bar (100 μm) in A also applies to B, C. Scale bar (100 μm) in D also applies to E–N.

Outside the normal locale of the retina, retina-like tissue and/or RPE-like tissue was detected in the optic nerve and/or in the vicinity of the optic nerve region, in around 30% of the eyes with photoreceptor-like cells [32]. In the eye of a P19 P_RPE65_-ngn3 mouse, DAPI staining showed a piece of densely populated tissue, a histological mark of the retina, in the optic nerve that is otherwise loosely populated (Fig. 6B, E, H, M). The highly populated region contained cells positive for photoreceptor proteins recoverin (Fig. 6C) and red opsin and rhodopsin (Fig. 6F), proteins responsible for capturing photon and initiating phototransduction process. The tissue also contained cells positive for proteins of INL neurons, including Otx2 of bipolar cells (Fig. 6I), Pax6 (Fig. 6J) and AP2α (Fig. 6N) of amacrine cells. Like in the retina, there was no overlap of Otx2^+^ and Pax6^+^ cells in the retina-like tissue (Fig. 6K). Bright-field view showed the presence of highly melanized, RPE-like tissue in the region (Fig. 6A, D, G, L).

**Figure 7:**
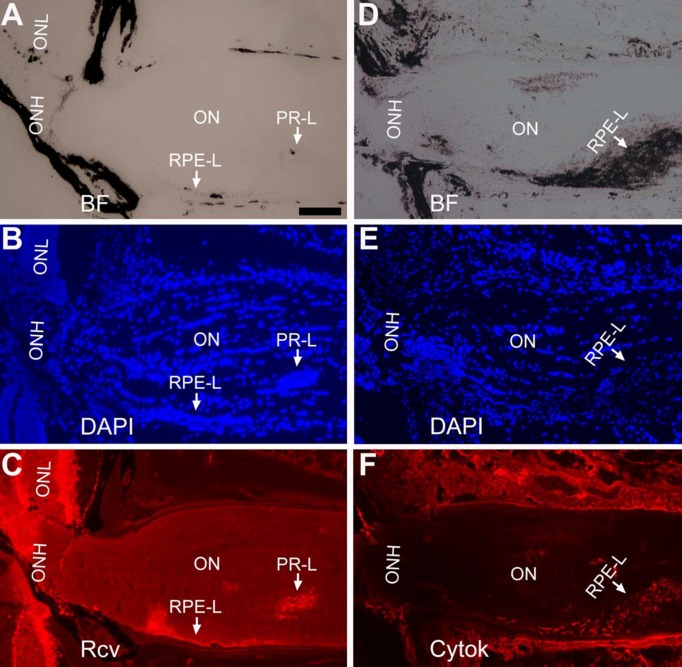
Photoreceptor-like cells and RPE-like cells in the optic nerve of P_VMD2_-ngn1 and P_RPE65_-ngn3 mice. A–C: Photoreceptor-like cells in a 2.3-month-old P_RPE65_-ngn3 mouse. A: Bright-field (BF). B: DAPI staining of the nuclei. C: Immunostaining for photoreceptor protein recoverin (Rcv). D–F: RPE-like cells in a 13-month-old P_VMD2_-ngn1 mouse. D: Bright-field (BF). E: DAPI staining of the nuclei. F: Immunostaining for cytokeratin 18 (Cytok), which is expressed in RPE cells. ON: optic nerve; ONH: optic nerve head; RPE-L: RPE-like. Scale bar (100 μm) applies to all panels.

Subsequent examination of adult (Fig. 7 A–C) and aged (Fig. 7 D–F) mice detected the presence of recoverin^+^ cells (Fig. 7 A–C) and cytokeratin 18^+^ cells (Fig. 7 D–F; RPE-like) in the optic nerve.

**Figure 8:**
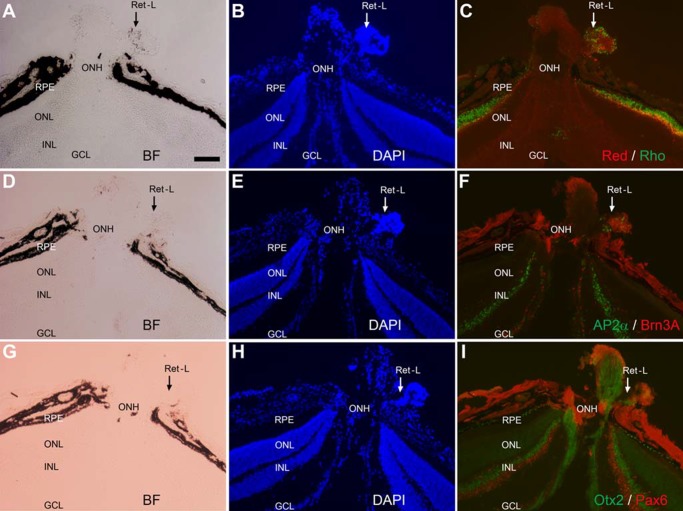
Retina-like tissue exterior to the eyecup and in the vicinity of the optic nerve head of a 5-week-old P_RPE65_-ngn3 mouse. A–C: Bright-field (A), DAPI staining (B), and double immunostaining (C) for red opsin (Red) and for rhodopsin (Rho, in green) of a posterior region of the eye. D–F: Bright-field (D), DAPI staining (E), and double immunostaining for AP2α (in green), a marker for horizontal cells and amacrine cells, and for Brn3A (in red), a marker for a subset of ganglion cells, on a serial section of the region. G–I: Bright-field (G), DAPI staining (H), and double immunostaining for a bipolar cell marker Otx2 (in green) and for an amacrine cell marker Pax6 (in red) of a serial section of the region. ON, optic nerve; ONH: optic nerve head; Ret-L: retina-like. Scale bar (100 μm) applies to all panels.

On rare occasions, retina-like tissue was detected in the vicinity of the optic nerve head (Fig. 8). Regions with densely populated cells (Fig. 8B, E, H) were positive for red opsin (Fig. 8C) and rhodopsin (Fig. 8C), bipolar protein Otx2 (Fig. 8I), amacrine proteins AP2α (Fig. 8F) and Pax6 (Fig. 6I), and ganglion protein Brn3A (Fig. 8F).

### Retina-like tissue in the choroid and the vicinity of the ciliary body

**Figure 9:**
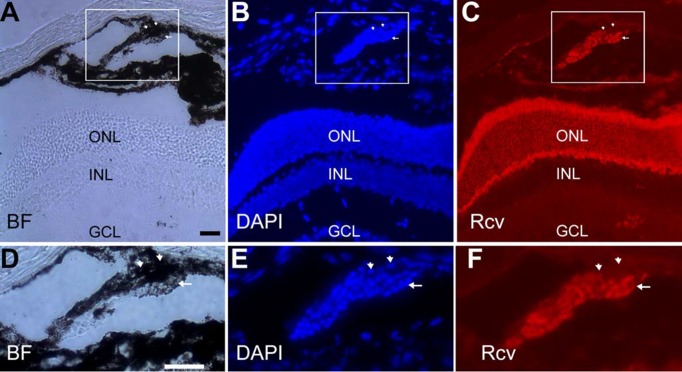
Photoreceptor-like cells in the choroid near the ciliary body of a 4-week-old P_VMD2_-ngn1 mouse. A: Bright-field (BF). B: DAPI staining of the nuclei. C: Immunostaining for photoreceptor protein recoverin (Rcv). D–F: Higher magnification of the boxed region in A–C. Arrowhead: Darkly pigmented cells that lacked recoverin expression. Arrow: recoverin^+^ cell with visible pigment granules. Scale bars (50 μm) in A also applies to B, C and that in D also applies to E, F.

On rare occasions, we observed the presence of retina-like tissue at places with no apparent direct connection with the neural retina. In a 4-week-old P_VMD2_-ngn1 mouse, a small stretch of cells expressing recoverin was detected in the choroid near the ciliary body (Fig. 9). The recoverin^+^ cells seemed attached to a layer of darkly pigmented cells. While the highly melanized cells at the region were negative for recoverin (arrowhead; Fig. 9 D–F), some of weakly melanized cells were recoverin^+^ (arrow; Fig. 9 D–F). These lightly pigmented, recoverin^+^ cells might be en-route to becoming photoreceptor-like from pigmented cells, as those in transitional stages in RPE-to-photoreceptor reprogramming described previously [32].

**Figure 10:**
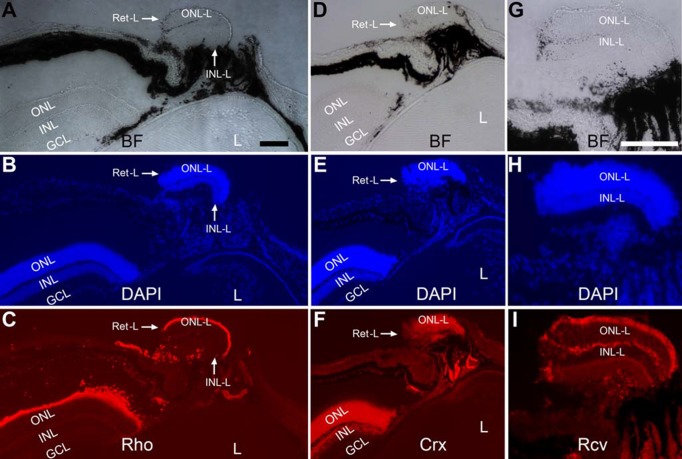
Retina-like tissue near the ciliary body in a 4-week-old P_VMD2_-ngn1. A–C: Bright-field (A), DAPI staining (B), and immunohistochemistry with anti-rhodopsin (Rho, C) of a cross-section of a region around the ciliary body. D–F: Bright-field (D), DAPI staining (E), and immunohistochemistry with anti-Crx (F) of a serial section of the same region. G–I: Bright-field (G), DAPI staining (H), and immunohistochemistry with anti-recoverin (Rcv, I) of another serial section of the same region. L, lens; Ret-L: retina-like; ONL-L: ONL-like; INL-L: INL-like. Scale bars (100 μm) in A also applies to B–F and that in G also applies to H, I.

In this animal, a small piece of retina-like tissue was present exteriorly near the ciliary body (Fig. 10). DAPI staining showed distinctive ONL-like and INL-like layers (Fig. 10B, E, H). Cells in the ONL-like were immunopositive for photoreceptor proteins rhodopsin (Fig. 10C), Crx (Fig. 10F), a transcription factor important for photoreceptor differentiation and function, and recoverin (Fig. 10I).

**Figure 11:**
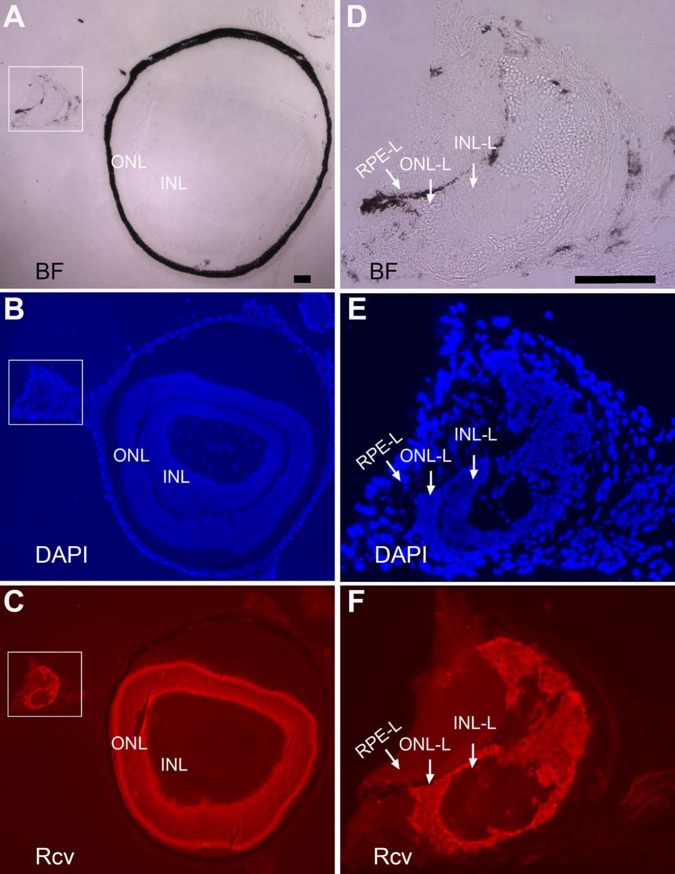
Retina-like tissue exterior to the eyecup. A–C: Bright-field view (A), DAPI staining of the nuclei (B), and immunostaining for photoreceptor protein recoverin (Rcv, C) of a cross-section of the eye from a P19 P_RPE65_-ngn3 mouse (a littermate of the one shown in Fig. 6). D–F: Higher magnification of the boxed region in A–C. Ret-L: retina-like; ONL-L: ONL-like; INL-L: INL-like. Scale bars (100 μm) in A also applies to B, C and that in D also applies to E, F.

In a littermate of the mouse with retina-like tissue present in the optic nerve (Fig. 6), we observed retina-like tissue exterior to the eyecup (Fig. 11 A–C). Like those at other ectopic locations, this retina-like tissue contained highly melanized tissue (RPE-like) and a demarcation of recoverin-positive, ONL-like layer and recoverin-negative, INL-like layer (Fig. 11 D–F). Although possible, it is unlikely that this ectopic tissue was a processing artifact, because all serial sections had been viewed and no contact of this structure with the retina was seen. On the other hand, this ectopic tissue could possibly have originated from the vicinity of the optic nerve head region, as this animal was a littermate of those with retina-like tissue in the optic nerve.

## Discussion

Retinal regeneration in the mammalian eye remains an elusive goal. In these experiments we showed the presence in P_VMD2_-ngn1 and P_RPE65_-ngn3 transgenic mice of tissues that displayed histological and molecular properties of the retina. The mouse retina consists of 3 nuclear layers, with the ONL being the thickest and the GCL the thinnest. These histological feature were displayed by the retina-like tissues, albeit sometimes a GCL-like layer was undetectable. The absence of GCL-like layer could be an underrepresentation due to its small size (thickness). It could also be a result of cell death from inflammation, reactive gliosis, lacking trophic supports normally derived from postsynaptic targets but absent for the retinal-like tissue, and lacking oxygen supply to maintain all retinal layers due to diminished blood vessels. Like in the retina, cells in the ONL-like layer expressed Crx, recoverin, red opsin, and rhodopsin, cells in the INL-like layer were positive for Otx2, AP2α, and Pax6, and cells in the GCL-like layer were positive for Brn3A. These data suggest that retina-like tissue was induced by ectopic *ngn1* expression from the *Bestrophin1* promoter and by ectopic *ngn3* expression from the *RPE65* promoter.

Anatomically, the retina-like tissue localized both within and outside the normal locale of the retina. There was substantial occurrence of retinal-like tissue present in the optic nerve region. It is possible that these retina-like tissue had originated from the retina and had migrated and/or extended into the optic nerve region. An alternative scenario is that it was produced from the optic nerve region, particularly the optic head region.

Rather remarkably, retina-like tissue at an ectopic location was detected in young and adult mice. In the adult animal, the tissue might be generated at a young age, including an embryonic stage, and survived well into adulthood. Alternatively, it was generated during adulthood. Either scenario is intriguing and warrants corroboration with further investigation. Perhaps equally interesting and important, the retina-like tissue was often associated with RPE-like tissue. Considering RPE’s importance in retina’s function and health, the coexistence of retina-like and RPE-like tissues enhances the possibility of exploring this reprogramming strategy in inducing functional, retinal regeneration in the mammalian eye. Keep in mind, however, functional regeneration also requires successful establishment of blood supply to the regenerated retina and integration of the new one into the existing visual circuitry.

Intriguing as they may seem, our data raise more questions than they answered. The two key ingredients in this study were proneural gene *ngn1* or *ngn3* and the cells that expressed the transgene under P_VMD2_ or P_RPE65_; neither is well understood. The selection of *ngn1* and *ngn3* was based on previous studies with the chick system. *Ngn1* and *ngn3* are found to be the frontrunners among more than 20 genes and factors tested in terms of efficiently reprogramming RPE cells to differentiate into cells displaying photoreceptor traits [30]. In the chick retina, *ngn1* and *ngn3* are transiently expressed in early developmental stages, when active cell proliferation and cell fate specification take place [43,44]. Gain-of-function studies suggest that *ngn3* promotes early retinal neurogenesis, and *ngn1* is one of the downstream genes of *ngn3* [43]. Currently, little is known about whether and how *ngn1* and *ngn3* participate in retinal neurogenesis in the mouse retina.

Despite of the design that aimed at targeting the transgene expression in differentiated RPE cells, the RPE might not be the source, at least not the sole source, of the ectopic retinal-like tissue. The transgenic mice analyzed in this study were created with the simple P_VMD2_-ngn1 and P_RPE65_-ngn3 DNA constructs, which lacked additional temporal and spatial regulatory elements for a tighter regulation of the transgene. Thus, the levels, the locales, and time of the transgene expression might have varied substantially. In addition, current information does not rule out the expression of *Bestrophin1* and *RPE65* outside the RPE, including in the developing retina and in Müller glia [45], which has been shown to maintain certain progenitor properties and to give rise to neuronal cells under proper conditions. Müller glia appeared normally present in eyes with photoreceptor-like cells in the subretinal space, suggesting either Müller glia was not a significant source of the photoreceptor-like cells or Müller glia had regenerated itself in case some of its cells had taken on the path to photoreceptor. Activation (as indicated by strong GFAP immunoreactivity) and atrophy (as indicated by diminished GS and CRALBP immunoreactivity) of Müller glia were apparent at place dismal to the RPE in the eye with two co-existing retinae, heightening the importance of native environment to retinal health.

The scenario of RPE as one, although not necessarily the only, source of the retina-like tissue is consistent with published data. Stem-like cells with multi-potentials have been isolated from the RPE of human adults [46]. Cells seemingly amid RPE-to-photoreceptor transformation have been found in young and old P_VMD2_-ngn1 and P_RPE65_-ngn3 transgenic mice, and eyecup explants of “sclera+choroid+RPE” from adult P_VMD2_-ngn1 and P_RPE65_-ngn3 animals can generate, de novo, photoreceptor-like cells in culture [32], providing direct support to the RPE giving rise to new photoreceptor-like cells.

When located in the subretinal space, the retinal-like tissue might displayed a reversed tissue polarity in regarding to the RPE, as observed in the classic RPE-to-retina transdifferentiation. However, unlike the classic RPE-to-retina transdifferentiation where the new retina is produced at the expense of the RPE, the RPE remained in our transgenic mice, as previously shown [32]. Furthermore, melanized tissues were often co-existed with the retina-like tissue regardless of the location. This implies the RPE might have the potential to give rise to both RPE cells (wound healing) and retinal cells under proper guidance.

The presence of retina-like tissue at ectopic locations, particularly in the optic nerve, the vicinity of optic nerve head, the choroid, and exterior to the eyecup and the co-presence of RPE-like tissue with retina-like tissue imply the presence of progenitor-like cells, including those embedded inside the RPE. These progenitor-like cells had responded to *ngn1* and *ngn3* expression from P_VMD2_ and P_RPE65_ and had given rise to the new tissues. No information is available on the identities of these cells and whether they intrinsically remained multi-potential progenitor properties or such properties were bestowed by ectopic expression of *ngn1* and *ngn3*. In-depth investigation with a wide scope is needed for a better understanding of the process before applying the strategy for retinal regeneration in the mammalian eye.

## References

[1] Kubota R, Hokoc JN, Moshiri A, McGuire C, Reh TA (2002) A comparative study of neurogenesis in the retinal ciliary marginal zone of homeothermic vertebrates. Brain Res Dev Brain Res 134: 31–41. 10.1016/S0165-3806(01)00287-5 11947935

[2] Ohta K, Ito A, Tanaka H (2008) Neural stem/progenitor cells in the vertebrate eye. Develop Growth Differ 50: 253–259. 10.1111/j.1440-169X.2008.01006.x 18336580

[3] Tropepe V, Coles BLK, Chaisson BJ, Horsford DJ, Elia AJ, et al. (2000) Retinal stem cells in the adult mouse eye. Science 287: 2032–2036. 10.1126/science.287.5460.2032 10720333

[4] Ahmad I, Tang L, Pham H (2000) Identification of neural progenitors in the adult mammalian eye. Biochem Biophys Res Commun 270: 517–521. 10.1006/bbrc.2000.2473 10753656

[5] Cicero SA, Johnson D, Reyntjens S, Frase S, Connell S, et al. (2009) Cells previously identified as retinal stem cells are pigmented ciliary epithelial cells. Proc Natl Acad Sci USA 106: 6685–6690. 10.1073/pnas.0901596106 19346468 PMC2672506

[6] Gualdoni S, Baron M, Lakowski J, Decembrini S, Smith AJ, et al. (2010) Adult ciliary epithelial cells, previously identified as retinal stem cells with potential for retinal repair, fail to differentiate into new rod photoreceptors. Stem Cells 28: 1048–1059. 10.1002/stem.423 20506130

[7] Al-Hussaini H, Kam JH, Vugler A, Semo M, Jeffery G (2008) Mature retinal pigment epithelium cells are retained in the cell cycle and proliferate in vivo. Mol Vis 14: 1784–1791. 18843376 PMC2562424

[8] Machemer R, Lauqa H (1975) Pigment epithelium proliferation in retinal detachment (massive periretinal proliferation). Am J Ophthalmol 80; 1–23. 808131 10.1016/0002-9394(75)90862-4

[9] Lauqa H, Machemer R (1975) Clinical-pathological correlation in massive periretinal proliferation. Am J Ophthalmol 80; 913–929. 811122 10.1016/0002-9394(75)90289-5

[10] Anderson DH, Stern WH, Fisher SK, Erickson PA, Borgula GA (1981) The onset of pigment epithelial proliferation after retinal detachment. Invest Ophthalmol Vis Sci 21: 10–16. 7251293

[11] Zhang NL, Samadani EE, Frank RN (1993) Mitogenesis and retinal pigment epithelial cell antigen expression in the rat after krypton laser photocoagulation. Invest Ophthalmol Vis Sci 34: 2412–2424. 8325749

[12] Rakoczy PE, Zhang D, Robertson T, Barnett NL, Papadimitriou J, et al. (2002) Progressive age-related changes similar to age-related macular degeneration in a transgenic mouse model. Am J Pathol 161: 1515–1524. 10.1016/S0002-9440(10)64427-6 12368224 PMC1867306

[13] Kiilgaard JF, Prause JU, Prause M, Scherfig E, Nissen MH, et al. (2007) Subretinal posterior pole injury induces selective proliferation of RPE cells in the periphery in in vivo studies in pigs. Invest Ophthalmol Vis Sci 48: 355–360. 10.1167/iovs.05-1565 17197554

[14] la Cour M (2008) ACTA-EVER lecture 2007. The retinal pigment epithelium: friend or foe? Acta Ophthalmol 86: 593–597. 10.1111/j.1755-3768.2008.01373.x 18752514

[15] Lopez PF, Yan Q, Kohen L, Rao NA, Spee C, et al. (1995) Retinal pigment epithelial wound healing in vivo. Arch Ophthalmol 113: 1437–1446. 10.1001/archopht.1995.01100110097032 7487607

[16] Del Priore LV, Hornbeck R, Kaplan HJ, Jones Z, Valentino TL, et al. (1995) Débridement of the pig retinal pigment epithelium in vivo. Arch Ophthalmol 113: 939–944. 10.1001/archopht.1995.01100070113034 7605288

[17] Ozaki S, Kita M, Yamana T, Negi A, Honda Y (1997) Influence of the sensory retina on healing of the rabbit retinal pigment epithelium. Curr Eye Res 16: 349–358 10.1076/ceyr.16.4.349.10696 9134324

[18] Sugino IK, Wang H, Zarbin MA (2003) Age-related macular degeneration and retinal pigment epithelium wound healing. Mol Neurobiol 28: 177–194. 10.1385/MN:28:2:177 14576455

[19] Rabenlehner D, Stanzel BV, Krebs I, Binder S, Goll A (2008) Reduction of iatrogenic RPE lesions in AMD patients: evidence for wound healing? Graefes Arch Clin Exp Ophthalmol 246: 345–352. 10.1007/s00417-007-0658-6 17704936

[20] Crisanti S, Guidry C (1995) Transdifferentiation of retinal pigment epithelial cells from epithelial to mesenchymal phenotype. Invest Ophthalmol Vis Sci 36: 391–405. 7531185

[21] Zhao S, Thornquist SC, Barnstable CJ (1995) In vitro transdifferentiation of embryonic rat pigment epithelium to neural retina. Brain Res 677: 300–310. 10.1016/0006-8993(95)00163-K 7552256

[22] Sakami S, Etter P, Reh TA (2008) Activin signaling limits the competence for retinal regeneration from the pigmented epithelium. Mech Dev 125: 106–116. 10.1016/j.mod.2007.10.001 18042353 PMC2254174

[23] Bumsted KM, Barnstable CJ (2000) Dorsal retinal pigment epithelium differentiates as neural retina in the microphthalmia (mi/mi) mouse. Invest Ophthalmol Vis Sci 41: 903–908. 10711712

[24] Nguyen M, Arnheiter H (2000) Signaling and transcriptional regulation in early mammalian eye development: a link between FGF and MITF. Development 127: 3581–3591. 10903182 10.1242/dev.127.16.3581

[25] Martínez-Morales JR, Dolez V, Rodrigo I, Zaccarini R, Leconte L, et al. (2003) OTX2 activates the molecular network underlying retina pigment epithelium differentiation. J Biol Chem 278: 21721–21731. 10.1074/jbc.M301708200 12663655

[26] Bäumer N, Marquardt T, Stoykova A, Spieler D, Treichel D, et al. (2003) Retinal pigmented epithelium determination requires the redundant activities of Pax2 and Pax6. Development 130: 2903–2915. 10.1242/dev.00450 12756174

[27] Bassett EA, Williams T, Zacharias AL, Gage PJ, Fuhrmann S, et al. (2010) AP-2alpha knockout mice exhibit optic cup patterning defects and failure of optic stalk morphogenesis. Hum Mol Genet 19: 1791–1804. 10.1093/hmg/ddq060 20150232 PMC2850623

[28] Fujimura N, Taketo MM, Mori M, Korinek V, Kozmik Z (2009) Spatial and temporal regulation of Wnt/beta-catenin signaling is essential for development of the retinal pigment epithelium. Dev Biol 334: 31–45. 10.1016/j.ydbio.2009.07.002 19596317

[29] Bharti K, Gasper M, Ou J, Brucato M, Clore-Gronenborn K, et al. (2012) A regulatory loop involving PAX6, MITF, and WNT signaling controls retinal pigment epithelium development. PLoS Genet 8:e1002757 10.1371/journal.pgen.1002757 22792072 PMC3390378

[30] Yan RT, Liang L, Ma W, Li X, Xie W, et al. (2010) Neurogenin1 effectively reprograms cultured chick retinal pigment epithelial cells to differentiate toward photoreceptors. J Comp Neurol 518: 526–546. 10.1002/cne.22236 20029995 PMC2927132

[31] Yan RT, Li X, Huang J, Guidry C, Wang SZ (2013) Photoreceptor-like cells from reprogramming cultured mammalian RPE cells. Mol Vis 19: 1178–1187. 23734087 PMC3669535

[32] Yan RT, Li X. Wang SZ (2013) Photoreceptor-like cells in transgenic mouse eye. Invest Ophthalmol Vis Sci 54: 4766–4775. 10.1167/iovs.13-11936 23847312 PMC3719446

[33] Halder G, Callaerts P, Gehring WJ (1995) Induction of ectopic eyes by targeted expression of the eyeless gene in Drosophila. Science 267: 1788–1792. 10.1126/science.7892602 7892602

[34] Chow RL, Altmann CR, Lang RA, Hemmati-Brivanlou A (1999) Pax6 induces ectopic eyes in a vertebrate. Development 126: 4213–4222. 10477290 10.1242/dev.126.19.4213

[35] Loosli F, Winkler S, Wittbrodt J (1999) Six3 overexpression initiates the formation of ectopic retina. Genes Dev 13: 649–654. 10.1101/gad.13.6.649 10090721 PMC316553

[36] Bernier G, Panitz P, Zhou X, Hollemann T, Gruss P, et al. (2000) Expanded retina territory by midbrain transformation upon overexpression of Six6 (optix2) in Xenopus embryos. Mech Dev 93: 59–69. 10.1016/S0925-4773(00)00271-9 10781940

[37] Lagutin O, Zhu CC, Furuta Y, Rowitch DH, McMahon AP, et al. (2001) Six3 promotes the formation of ectopic optic vesicle-like structures in mouse embryos. Dev Dyn 221: 342–349. 10.1002/dvdy.1148 11458394

[38] Coulombre JL, Coulombre AJ (1965) Regeneration of neural retina from the pigmented epithelium in the chick embryo. Dev Biol 12: 79–92. 10.1016/0012-1606(65)90022-9 5833111

[39] Bakall B, Marmorstein LY, Hoppe G, Peachey NS, Wadelius C, et al. (2003) Expression and localization of bestrophin during normal mouse development. Invest Ophthalmol Vis Sci 44: 3622–3628. 10.1167/iovs.03-0030 12882816

[40] Hamel CP, Tsilou E, Harris E, Pfeffer BA, Hooks JJ, et al. (1993) A developmentally regulated microsomal protein specific for the pigment epithelium of the vertebrate retina. J Neurosci Res 34: 414–425. 10.1002/jnr.490340406 8474143

[41] Park CM, Hollenber MJ (1989) Basic fibroblast growth factor induces retinal regeneration in vivo. Dev Biol 134: 201–205. 10.1016/0012-1606(89)90089-4 2731647

[42] Vergara MN, Del Rio-Tsonis K (2009) Retinal regeneration in the Xenopus laevis tadpole: a new model system. Mol Vis 15: 1000–1013. 19461929 PMC2684558

[43] Ma W, Yan R-T, Mao W, Wang S-Z (2009) Neurogenin3 promotes early retinal neurogenesis. Mol Cell Neurosci 40: 187–198. 10.1016/j.mcn.2008.10.006 19028584 PMC2659499

[44] Yan RT, He L, Wang SZ (2009) Pro-photoreceptor activity of chick neurogenin1. Invest Ophthalmol Vis Sci 50: 5567–5576. 10.1167/iovs.09-3647 19578021 PMC2788661

[45] Zhu M, Zheng L, Ueki Y, Ash JD, Le YZ (2010) Unexpected transcriptional activity of the human VMD2 promoter in retinal development. Adv Exp Med Biol 664: 211–216. 10.1007/978-1-4419-1399-9_24 20238019

[46] Salero E, Blenkinsop TA, Corneo B, Harris A, Rabin D, et al. (2012) Adult human RPE can be activated into a multipotent stem cell that produces mesenchymal derivatives. Cell Stem Cell 10: 88–95. 10.1016/j.stem.2011.11.018 22226358

